# The Ethics of Controversy

**Published:** 1919-05-03

**Authors:** 


### THE MATRONS' AND SISTERS' DEPARTMENT.

### THE ETHICS OF CONTROVERSY.

It is always a question whether it is worth while to expose the calculated "inexactitudes" which appear from time to time in the columns of the *British Journal of Nursing*, for, as "Ierne" explained last week of the Hydra (see The Hospital, April 26, page 88), for every head cut off two fresh ones sprout in its place. As, however, these darkenings of knowledge may be used to obscure the true issues before the eyes of Parliament we have decided to deal with some characteristic statements which appeared above its Editor's signature last Saturday.

Mrs. Bedford Fenwick alleges that there was "a determined attempt on the part of the representatives of the College of Nursing, Ltd., to most unjustly exclude" the nurses' organisations affiliated to the Central Committee "from representation in their own Bill." What are the facts? In the College Bill the Provisional Nursing Council consisted of thirty-six members, of whom twelve were to be appointed by the College and twelve by the Central Committee, an equal number of representatives from both, though the College has at least twice, and probably three times, as many trained nurses on its Register as the Central Committee can muster, all told, amongst its various "affiliated organisations." The real point of difference is that the Central Committee seeks to keep its nominees in office for two years in which to compile not only the first legal Register but also all the "rules" under which nurses, if registered, will have to live and practise, whereas the College Bill seeks to be rid of this non-elected Council as soon as possible when it has carried through the scheme for setting up the permanent Nursing Council with its two-thirds majority elected by the nurses themselves. Is it the College Bill or the Central Committee's Bill which can fairly be said to be "designed to suppress, rather than encourage, the evolution of the nursing profession."

The question of supplementary registers again affords a good illustration of Mrs. Bedford Fenwick's controversial methods. An attempt, which can only be described as impudent, is made to cast upon the College the odium of instituting a children's register—a proposition to which it may be remarked that not a word was said in opposition by the Central Committee's representatives—by asserting that "such a provision was made possible in Clause 4 of the College Bill. As though it were just the same thing for the Central Committee to swallow without even a grimace an amendment definitely setting up a register for children's nurses and for the College in its Bill to permit the formation of such a register, always provided that the General Nursing Council, with the majority of its members elected by the nurses on the general register, saw fit to recommend it to the Privy Council.

The paragraph headed "An Unjustifiable Pledge" may next claim some examination. Nurses are invited to join the College, amongst other reasons, "because the Council of the College of Nursing has drafted a 'Nurses' Registration Bill' which provides that the Register already formed by the College of Nursing shall be the first Register under the Act. If, therefore, you are on the College Register you will, automatically and without further fee, be placed upon the State Register when the Nurses' Registration Bill is passed." There is here no question of any Registration Bill except the Bill promoted by the College; obviously the Council could give no pledge as to any other Bill for the Registration of Nurses which might come before Parliament, and this fact has been pointed out time and time again by the *British Journal of Nursing* in its attempts to keep nurses out of the College. How, therefore, if the Central Committee's Bill should become law, the College could be "compelled to pay not only the 13,000 guineas paid to it by the nurses, but also any excess charged above £1 1s. for State Registration," it would puzzle the versatile Editor to explain, though quite characteristically she takes occasion to allude to a "financial imbroglio" existing only in her own imagination, and once more drags in "Lady Cowdray and her Committee," who, we make bold to say, have no more intention of using the Nation's Fund to pay registration fees than to employ it to defray the expenses of promoting the Central Committee's Bill.

What, however, may give the candid reader some amusement, if he is not tired of tracing the devious methods of the Central Committee or its supporters, is to observe the desperate struggle Mrs. Bedford Fenwick makes to circumvent the difficulties of finance, which from the first every one knew would be likely to wreck the Central Committee's Bill, unless they could obtain, as the College of Nursing has done, and as every University has had to do, adequate endowments. As the first College leaflet sent to members of Parliament asserted, no educational body can pay its way on fees alone. And the way of the Central Committee's Bill is a very expensive way. As Mrs. Bedford Fenwick admits, there will be three offices with three sets of officials to be kept up; the Annual Register will cost £3,000 a year; there will be highly qualified examiners to be paid in "anatomy, physiology, the theory of general medical and surgical nursing, including therapeutics, clinical nursing, gynæcological, infectious, children's, and other special branches of nursing, to say nothing of dietetics." And let us not forget, though upon this point Mrs. Bedford Fenwick lays no stress, that under the Central Committee's Bill "there shall be paid to members of the Council—forty-two of them ultimately, thirty-one for the first two years—such fees for attendance and travelling expenses as the Privy Council may allow." No wonder she feels constrained to add, with a final touch of exasperation, "When people begin to pettifog on the question of State Registration finance we have got to pray for patience without end."

